# An Individual Meta‐Analysis of Psychological Treatments for Mood and Personality Disorders Using the Severity Diagnostic System of ICD‐11

**DOI:** 10.1002/pmh.70110

**Published:** 2026-10-22

**Authors:** Min Yang, Peter Tyrer, Mike Crawford, Mary McMurran

**Affiliations:** ^1^ West China School of Public Health University of Sichuan Chengdu China; ^2^ Swinburne University of Technology Faculty of Life and Social Sciences Melbourne Australia; ^3^ Division of Psychiatry, Department of Brain Sciences Imperial College London UK; ^4^ Faculty of Social Sciences Nottingham Trent University Nottingham UK; ^5^ Cardiff School of Sport & Health Sciences Cardiff Metropolitan University Cardiff UK

## Abstract

An individual patient meta‐analysis was carried out to investigate the impact of the four levels of severity of ICD‐11 personality disorder on the short‐term outcome (12–24 m) of anxiety and depressive symptoms and social function in 1384 patients included in four randomised trials in which psychological treatments, mainly linked to cognitive‐behaviour therapy, were compared with standard controls. In each of these trials, a proxy form of the ICD‐11 classification system was used to assess personality status. The results showed that in those with more severe personality pathology, mood symptomatology was greater and showed a consistent linear trend with normal personality showing the least pathology and severe personality the most. Improvement in anxiety symptoms was significantly less in those with moderate and severe personality disorder compared with those with no personality disorder. Depressive symptoms did not show the same changes except in one trial. Social function improved in all patients in the studies. Treatment effects were small and not significantly different. The findings give some support to the dimensional system of classifying personality disorder and confirmed that all levels of personality dysfunction, including the sub‐syndromal condition of personality difficulty, have an impact on mental health symptomatology and function. Suggestions for improving the utility of the classification are made. In particular, the domain structure appears to have a major influence on clinical outcomes.

## 1 Introduction

Until recently, the assessment of treatments for personality disorder has been focused on individual categories such as borderline and antisocial personality disorder. Reviews have generally shown that drug treatment is ineffective but focused psychological treatments are of value, although the benefits still need to be more fully assessed (Choi‐Kain et al. 2017; Storebø et al. 2020; Stoffers‐Winterling et al. 2022; Crotty et al. 2024). Other personality disorders have generally been neglected in research studies even though the epidemiological evidence shows that they constitute the majority of those with personality disorder (Volkert et al. 2018; Winsper et al. 2020).

Since the ICD 11 revision of classification of personality disorder was first published (Tyrer et al. 2011), there has been considerable interest in the implications of the classification with regard to treatment. The main difference between the classification of personality disorder in ICD 11 and ICD 10 is the change from a categorical system to a dimensional one. The separation of personality difficulty, mild, moderate and severe personality disorder from no personality dysfunction is based on severity of disturbance and is likely to be relevant to the selection and intensity of treatment. There have been attempts to assess the impact of severity of disorder even with the old classification system. Some studies have suggested that severity has no influence on the outcome of psychological treatment (Kvarstein et al. 2019; Seow et al. 2020) whereas others have suggested more severe or persistent personality disorder leads to a worse outcome (Tyrer et al. 2016).

Although the ICD‐11 classification was only approved formally by the World Health Assembly in 2019, the essential elements of the dimensional system had been used in several studies many years earlier (Tyrer, Cooper, et al. 2014). Four large randomised trials of psychological treatments for mood and personality disorder have used this approach and are the subject of this individual patient meta‐analysis. Whilst substantial studies of the outcome of the ICD‐11 system are still awaited this investigation indicates some aspects that will need to be addressed.

This study asked three research questions: (1) Are the clinical profiles of anxiety and depressive disorders worse in those with more severe personality disturbance? (2) Do psychological treatments for anxiety and depression have a worse outcome in those with more severe personality disturbance? (3) Are the severity levels of personality disturbance described in ICD‐11 supported by outcome data (i.e., is there a graded response linked to incrementally greater severity)?

## 2 Method

All randomised trials of psychological treatments for either mood or personality disorders that recorded personality status using the principles of ICD‐11 were included in the study. Four studies were identified from a search of the literature of controlled trials of personality disorder that had measured recordings of severity and the individual patient data obtained for each of these.

1. The Nottingham Study of Neurotic Disorder (NSND), which randomised patients presenting with anxiety and depression to cognitive‐behaviour therapy (CBT), self‐help or drug treatments (Tyrer et al. 1988). The patients who received drug treatments in the NSND trial were not included in the individual meta‐analysis.
2. The treatment of recurrent self‐harm by brief cognitive therapy (POPMACT) (Tyrer et al. 2003).
3. The treatment of pathological health anxiety with modified CBT (CHAMP) (Tyrer, Cooper, et al. 2014; Sanatinia et al. 2016).
4. A trial of problem solving therapy combined with education for those presenting with personality disorder (PEPS) (McMurran et al. 2017). These studies involved a total of 1384 patients, most of whom were assessed using the same rating scales for anxiety and depression (the Hospital Anxiety and Depression Scale [HADS]) (Zigmond and Snaith 1983) and the Social Functioning Questionnaire (SFQ) (Tyrer et al. 2005).

### 2.1 Determination of Personality Status

All these studies were carried out before the ICD‐11 classification was approved for use, but they used a similar dimensional approach created earlier that separated personality dysfunction into different levels of severity (Tyrer, Cooper, et al. 2014), which allows a proxy equivalent of the ICD‐11 classification to be examined.

In the Nottingham Study, the baseline personality assessment was carried out with the Personality Assessment Schedule (PAS) (Tyrer et al. 1979). This schedule records both the severity and four levels of personality status (Tyrer et al. 1990). These were (1) no personality dysfunction; (2) personality difficulty, a score just below the level of personality disorder, (3) simple or mild personality disorder, with abnormal scores in one cluster domain only and (4) complex personality disorder, with scores for disorder in two or more cluster domains. A fifth group, those with severe personality disorder with additional antisocial pathology, was also recorded in some studies but the numbers were too low to analyse separately and so these were combined with complex personality disorder. An initial study was carried out with the PAS data being made available to two independent assessors who reassessed the personality status of each patients using the ICD‐11 criteria available at that time (Tyrer et al. 2011). A high level of agreement was found between the investigators (kappa = 0.93) (Tyrer, Crawford, et al. 2014). In the other three trials, a similar approach was used. In the POPMACT study (Tyrer et al. 2003; Tyrer et al. 2005) and the CHAMP trial (Tyrer, Cooper, et al. 2014; Sanatinia et al. 2016), a shortened version of the PAS, the Quick Personality Assessment Schedule (PAS‐Q), was used to record personality status and converted to ICD‐11 severity levels in the same way (Sanatinia et al. 2016). In the PEPS trial, personality disorder was recorded with the International Personality Disorder Examination (IPDE; Loranger et al. 1994) and included severity and domain assessments, but as all participants in the PEPS study had personality disorder, there was no third group without personality disturbance.

### 2.2 Statistical Analysis

The individual data of four randomised trials each had two arms, an active psychological treatment and a control group, with all having a baseline assessment of personality function, and with baseline scores of anxiety, depression and social function, and with all having repeated assessments up to 12 months after randomisation. To achieve matching with ICD‐11 personality status, the data were organized into four groups; no personality disturbance, personality difficulty, mild personality disorder, and moderate and severe personality disorder combined. This combination was made as two trials (POPMACT and NSND) did not separate the mild and severe PD in their raw data, and CHAMP trial had only a few cases in the severe level. PEPS did not include any individuals who had no personality disorder. The following statistical methods were used to investigate the research questions.

*Research Question 1*: did anxiety and depressive disorders show greater severity in those with more severe personality disturbance? For this analysis, we used the correlation measure, the Tau B coefficient. This measures the association between the continuous scale of clinical outcome and the ordinal PD severity level with ties. This analysis enables the raw correlation between the PD severity and the clinical outcomes to be estimated. We calculated the Tau B coefficient for each trial in the first stage, and then carried out meta regression analysis using the random effects model to summarise the measure in the second stage (Veroniki et al. 2023). This analysis did not adjust for covariates.

Because there could be potential confounding of sex and age of patients in influencing the correlation between the PD severity and the clinical outcomes, we further applied three level models (trial at Level 3, individual at Level 2, follow‐up time at Level 1). This model, taking into account the random effects among trial and among individuals, enabled us to test the association between PD severity and the outcome with adjustment for age and sex (Goldstein et al. 2000).

*Research Question 2*: are there differences in treatment outcomes at different levels of personality disturbance? To answer this question, we carried out a subgroup analysis by treatment group by first calculating the Tau B in the Stage 1 analysis and then testing the treatment effect in the meta‐regression analysis in Stage 2. We further tested the treatment effects on the PD and outcome association in the multilevel models with adjustment for age and sex. Based on the multilevel model analysis, we also tested the differences in change over time in the outcomes between treatment group and personality severity level. This was done by adding interaction terms between the treatment, the follow‐up time and PD severity in the models.

*Third research question*: What is the dose–response relationship between the level of PD severity and outcomes? This was done by testing the linearity of personality severity level and outcomes based on the final model estimates, with results presented in figures.

We used the R version 4.4.3 in calculating the Tau B coefficient and the confidence interval, MLwiN 3.03 for all modelling analysis and the random model meta‐analysis in the study.

## 3 Results

The baseline data for all four trials, confining the main measures to those outcomes that were common, are summarised (Table 1).

**TABLE 1:** Basic information of four trials at baseline and included in the analysis.

	Group	NSND	POPMACT	CHAMP	PEPS
N	Control	70	241	225	152
	Active	84	239	219	154
Age: mean (SD)	Control	36.26 (12.60)	31.24 (10.44)	47.00 (13.39)	37.95 (11.03)
	Active	36.34 (11.97)	31.73 (11.99)	50.29 (13.56)	38.78 (10.90)
Female: *N* (%)	Control	56 (80.0)	164 (68.0)	123 (54.7)	115 (75.7)
	Active	50 (59.5)	162 (67.8)	113 (51.6)	115 (74.7)
HADSD (depression): mean (SD)	Control	9.21 (4.47)	11.18 (4.19)	8.83 (4.58)	12.69 (4.65)
	Active	11.13 (4.30)	11.26 (4.95)	9.12 (4.34)	12.71 (4.24)
HADSA (anxiety): mean (SD)	Control	13.51 (3.76)	14.28 (3.59)	12.25 (3.88)	14.81 (3.84)
	Active	14.10 (3.66)	13.98 (4.27)	12.56 (3.74)	14.87 (3.72)
SFQ (social function): mean (SD)	Control	N/A	13.30 (4.26)	9.49 (4.32)	14.37 (4.48)
	Active	N/A	13.27 (4.88)	9.42 (3.74)	14.98 (4.03)
PD severity: *N* (%)^a^	Control				
	Lev. 1	32 (49.2)	18 (7.5)	34 (15.1)	N/A
	Lev. 2	11 (16.9)	122 (50.6)	97 (43.1)	30 (14.7)
	Lev. 3	15 (23.1)	61 (25.3)	74 (32.9)	59 (38.8)
	Lev. 4	7 (10.8)	40 (16.6)	20 (8.9)	63 (41.5)
	Active				
	Lev. 1	32 (40.0)	25 (10.5)	29 (13.2)	N/A
	Lev. 2	17 (21.2)	113 (47.3)	100 (45.7)	18 (11.7)
	Lev. 3	20 (25.0)	65 (27.2)	68 (31.1)	71 (46.1)
	Lev. 4	11 (13.8)	36 (15.1)	22 (10.1)	65 (42.2)

^a^ Lev. 1 for No PD, Lev. 2 for PD Difficult, Lev. 3 for Mild PD, Lev. 4 for Moderate and Severe PD.

The assessment of heterogeneity of the three outcome measures—depression anxiety and social function—showed considerable variation within and among the participants in the four trials but an acceptable degree of homogeneity between the trials with the test *p* value as 0.165 for depression, 0.166 for anxiety and 0.226 for social function (Table 2).

**TABLE 2:** Variation (τ^2^) of random effects among trials, among patients and within patients in whole sample (heterogeneity test).

Effect level	Depression		Anxiety		Social function	
	Variance (SE)	*p*	Variance (SE)	*p*	Variance (SE)	*p*
Among trials	2.65 (1.91)	0.165	2.03 (1.47)	0.166	4.39 (3.63)	0.226
Among patients	13.09 (0.66)	0.000	9.55 (0.52)	0.000	13.86 (0.72)	0.000
Within patient	11.74 (0.30)	0.000	11.83 (0.30)	0.000	9.98 (0.30)	0.000

### 3.1 Effect of Level of Personality Dysfunction on Outcomes

The level of personality severity had a significant influence on outcomes. Each increase in the level of the personality disorder was significantly associated with a worse outcome in all four trials, in different treatment groups and at different follow‐up times. Detailed statistical results are presented in the Tables S1 and S2. The dose–response relationship between level of personality dysfunction and clinical profiles of individuals with adjustment for age and sex, and separated for treatment group and follow‐up time, remained significant (Table 3 and Figure 1). The linear improvement in the outcomes from the most severe level of PD to no PD is clearly shown in Table 3 with *p* values for all outcomes smaller than 0.001, all these data supporting the dimensional classification of personality.

**TABLE 3:** Impact of PD severity on outcomes, adjusted for age and sex (Models A and C).

PD severity	Depression (*N* = 1387)		Anxiety (N = 1387)		Social function (*N* = 1230)	
	Mean difference (SE)	Sig. *p*	Mean difference (SE)	*p*	Mean difference (SE)	Sig. *p*
Model A						
Moderate/severe	Ref		Ref		Ref	
No PD ‐ Ref	−3.80 (0.42)	0.000	−2.87 (0.38)	0.000	−4.70 (0.49)	0.000
Difficulty ‐ Ref	−2.88 (0.32)	0.000	−2.17 (0.29)	0.000	−3.30 (0.34)	0.000
Mild ‐ Ref	−1.49 (0.32)	0.000	−0.75 (0.28)	0.009	−1.28 (0.33)	0.000
Linear trend test		0.000		0.000		0.000
Model B						
Active vs. Control	−0.45 (0.49)	0.357	−0.59 (0.44)	0.188	−0.10 (0.52)	0.840
Control group						
Moderate/severe	Ref		Ref		Ref	
No PD ‐ Ref	−4.54 (0.45)	0.000	−3.24 (0.52)	0.000	−5.06 (0.67)	0.000
Difficulty ‐ Ref	−2.93 (0.44)	0.000	−2.38 (0.40)	0.000	−3.18 (0.46)	0.000
Mild ‐ Ref	−1.65 (0.58)	0.000	−0.83 (0.41)	0.041	−1.35 (0.46)	0.003
Linear trend test		0.000		0.000		0.000
Active vs. Control group						
Active*No PD—Ref	1.44 (0.78)	0.064	0.71 (0.70)	0.310	0.72 (0.92)	0.832
Active*Difficulty ‐ Ref	0.08 (0.61)	0.896	0.40 (0.55)	0.461	−0.22 (0.63)	0.725
Active*Mild ‐ Ref	0.31 (0.62)	0.618	0.17 (0.56)	0.758	0.14 (0.65)	0.438
Linear trend test		0.112		0.401		0.503
Model C						
Follow‐up month	−0.08 (0.02)	0.000	−0.08 (0.02)	0.000	−0.04 (0.02)	0.013
Moderate/severe	Ref		Ref		Ref	
No PD ‐ Ref	−3.55 (0.46)	0.000	−2.27 (0.42)	0.000	−4.94 (0.54)	0.000
Difficulty ‐ Ref	−3.06 (0.35)	0.000	−2.04 (0.32)	0.000	−3.32 (0.37)	0.000
Mild ‐ Ref	−1.57 (0.35)	0.000	−0.74 (0.32)	0.020	−1.24 (0.37)	0.001
Linear trend test		0.000		0.000		0.000
Follow‐up month*PD						
Month*No PD ‐Ref	−0.026 (0.025)	0.292	−0.074 (0.024)	0.002	0.028 (0.029)	0.331
Month*Difficulty‐Ref	0.027 (0.021)	0.199	−0.016 (0.021)	0.450	0.003 (0.021)	0.885
Month*Mild‐Ref	0.013 (0.022)	0.537	−0.001 (0.021)	0.956	−0.005 (0.022)	0.822
Linear trend test		0.063		0.000		0.203

*Note:* Sig. *p* value came from generalized Ward Test.

**FIGURE 1:**
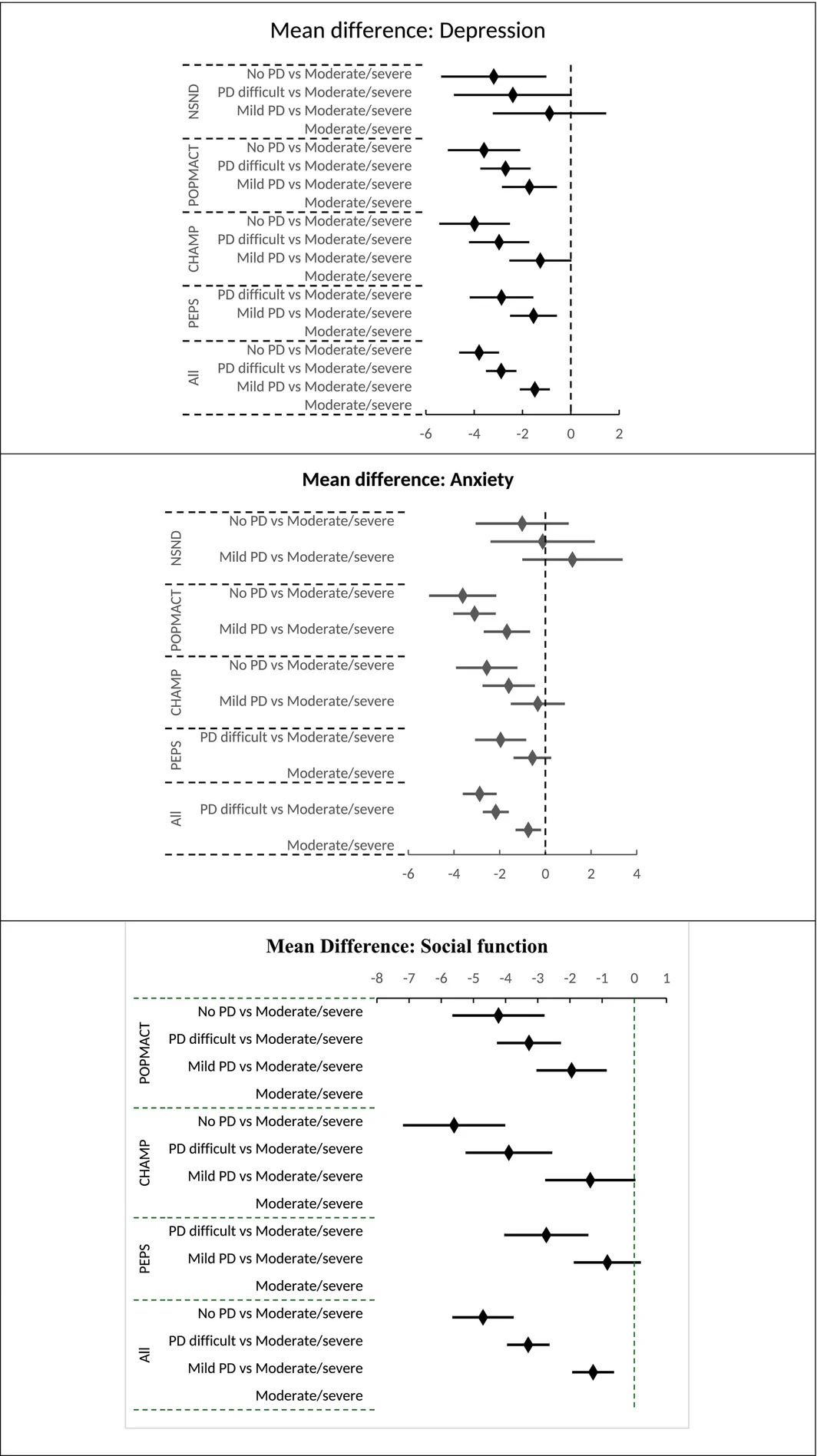
Mean difference in outcomes by PD severity based on Model A (the reference level was the moderate/severe at the value zero).

### 3.2 Effect of Treatment on Outcomes by Level of Personality Dysfunction

There was no difference in the association between the personality severity level and the outcomes of the treatment and control groups with Tau B coefficients as 0.161 (0.108–0.214) vs. 0.194 (0.149–0.239) for depression, 0.138 (0.086–0.190) vs. 0.173 (0.128–0.218) for anxiety and 0.208 (0.137–0.279) vs. 0.220 (0.145–0.294) for social function (S1). The age and sex adjusted model suggested that treatment effects were not statistically significant for any level of PD severity in all outcomes (Table 3). Based on the model estimate in Table 3, the calculated Cohen effect size by PD severity level was in the range of −0.05–0.23 for depression, −0.07–0.09 for anxiety and −0.03–0.14 for social function.

However, the rate of change in the analysis of outcomes by treatment indicated a faster decline of symptoms in the active group compared with the control group for anxiety (*p* = 0.013), a smaller decline in depression (*p* = 0.088) and no difference in social function (*p* = 0.827) (Table 3). Further time trend analysis of treatment group by personality level revealed that over time, individuals with no PD improved significantly more than all other PD levels for depression and anxiety. The individuals with higher levels of personality disturbance in the active group showed somewhat more improvement than those in the control group for depression and anxiety particularly, but this was not statistically significant (Figure 2).

**FIGURE 2:**
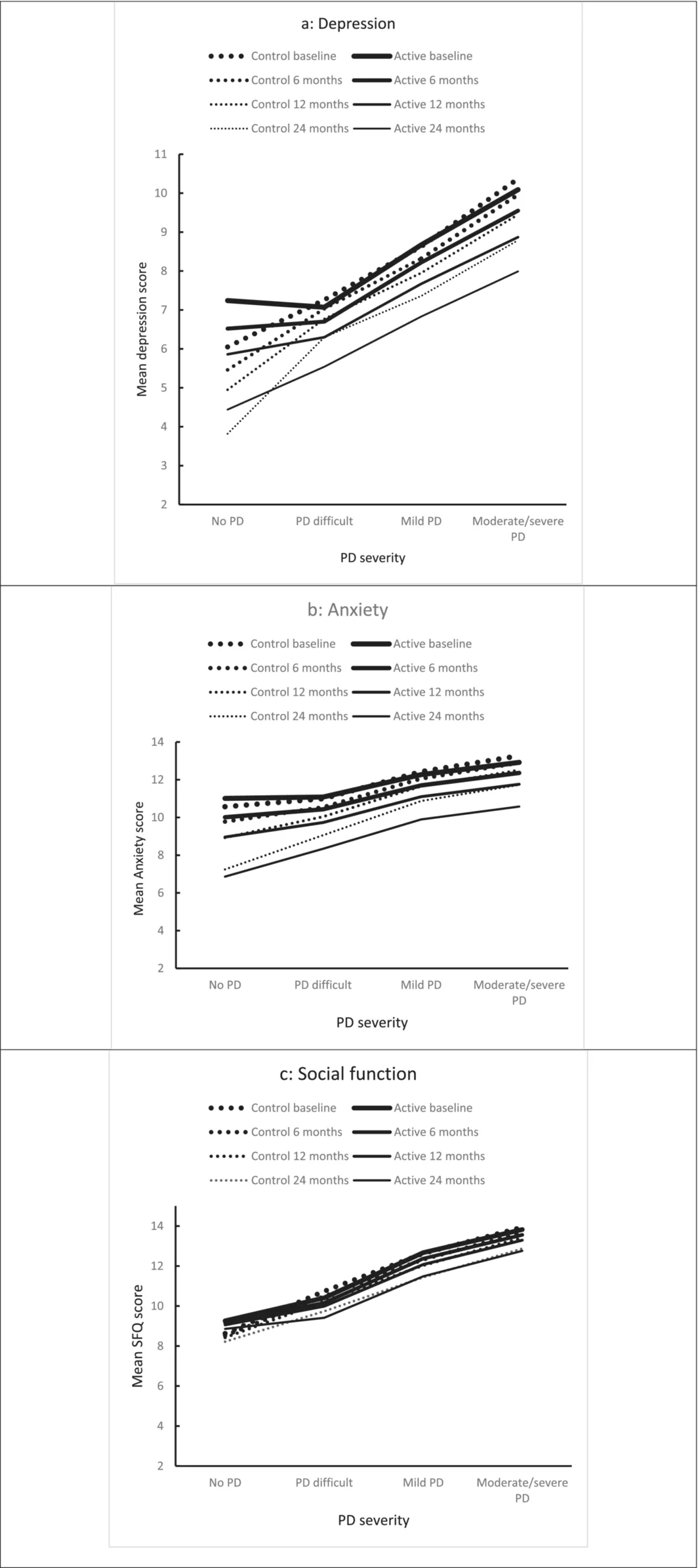
Estimated and adjusted association between PD severity and outcomes by treatment by follow‐up time.

### 3.3 Effect of Time on Outcomes by Level of Personality Dysfunction

In the modelling analysis of the study, we examined outcome data over the weeks up to 24 months and found a consistent decline over time in all outcomes with *p* = 0.001 for depression, 0.001 for anxiety and 0.021 for social function (Table 3). Disentangling the overall decline in time by level of PD severity in comparison with the moderate and severe level, it was found that the overall declining trend in anxiety was most marked (*p* = 0.002) with the largest decline in those with no personality dysfunction (−0.076; 95% CI: −0.123, 0.029), which then led to a significant linear change in the anxiety by severity level of personality disturbance overall (*p* = 0.000; Table 3). The clear difference in the change rate between those with no personality disturbance and moderate/severe PD can also be seen in Figure 2b and Table 4. The difference in the change rate among PD levels was marginal for depression (overall *p* = 0.07, linearity *p* = 0.056). No effect of time on social function by PD severity level was found (Figure 3).

**TABLE 4:** Monthly change rate of outcomes by PD severity and by treatment group, adjusted for age and sex (based on Model D).

	Depression (*N* = 1387)		Anxiety (*N* = 1387)		Social function (*N* = 1230)	
	Change rate/month (SE)	Sig. *p*	Change rate/month (SE)	Sig. *p*	Change rate/month (SE)	Sig. *p*
Control						
Time (in month)	−0.058 (0.026)	0.028	−0.070 (0.026)	0.007	−0.047 (0.025)	0.060
Time*moderate/severe PD	Ref		Ref		Ref	
Time*No PD ‐ Ref	−0.076 (0.036)	0.035	−0.118 (0.035)	0.000	−0.011 (0.040)	0.783
Time*Difficult ‐ Ref	0.015 (0.031)	0.629	−0.008 (0.031)	0.796	0.009 (0.030)	0.764
Time*Mild ‐ Ref	0.023 (0.032)	0.472	0.009 (0.031)	0.772	−0.012 (0.043)	0.780
Linear trend test		0.001		0.000		0.786
Active vs. Control						
Time (in month)	−0.042 (0.036)	0.239	−0.025 (0.035)	0.480	0.005 (0.036)	0.881
Time*moderate/severe PD	Ref		Ref		Ref	
Time*No PD ‐ Ref	0.096 (0.049)	0.050	0.085 (0.049)	0.083	0.078 (0.057)	0.171
Time*Difficult ‐ Ref	0.021 (0.043)	0.625	−0.049 (0.042)	0.244	−0.012 (0.043)	0.780
Time*Mild ‐ Ref	−0.018 (0.043)	0.676	−0.020 (0.043)	0.642	−0.027 (0.044)	0.540
Linear trend test		0.007		0.012		0.041

**FIGURE 3:**
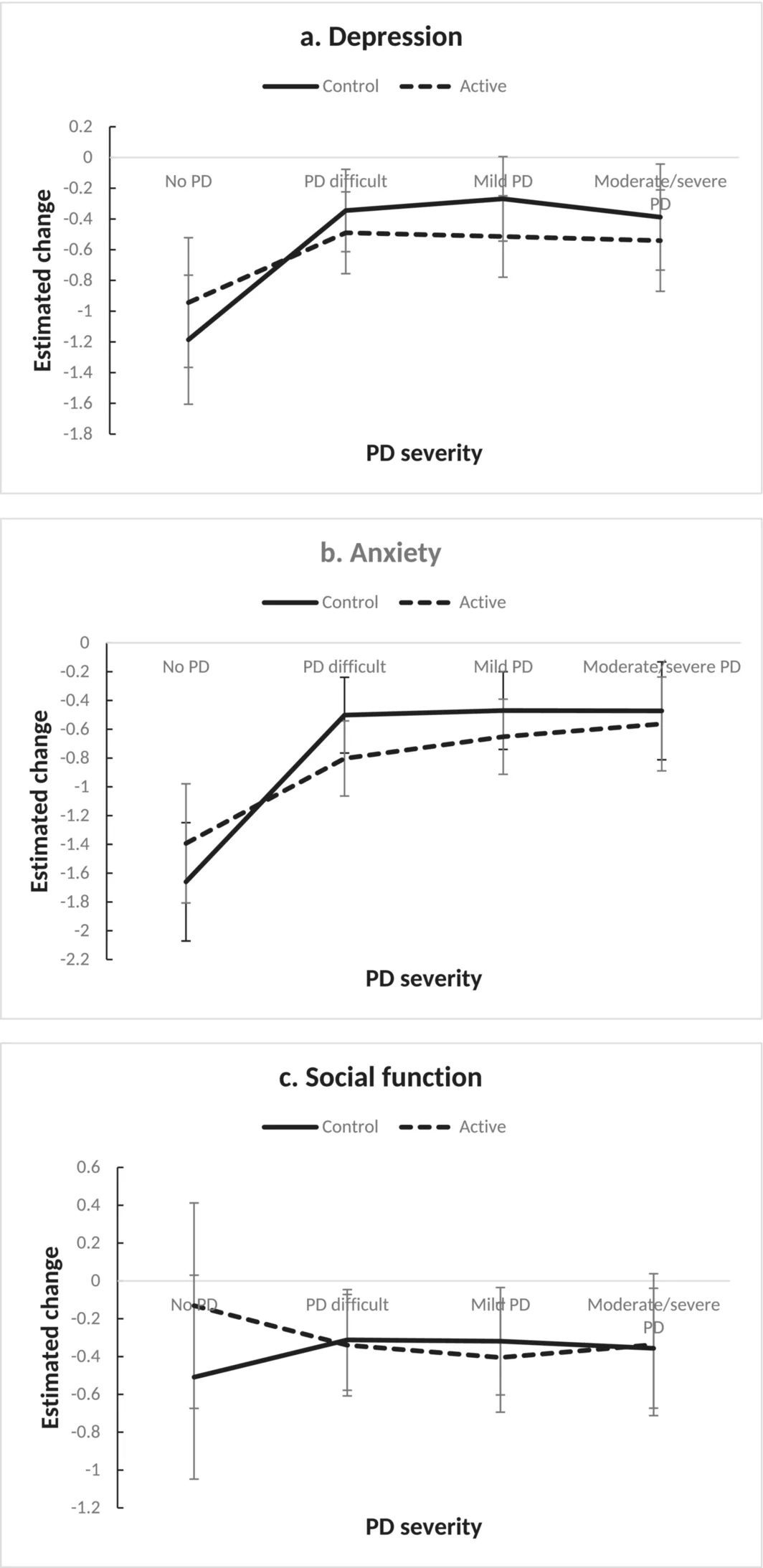
Estimated change rate of outcomes with 95% CI by personality severity by treatment group from Model D.

## 4 Discussion

The results of this meta‐analysis represent only the first stage of evaluating the potential value of the ICD 11 classification of personality disorders. The classification has not yet been implemented in most countries but this study illustrates some of the issues that may arise in its use. The main limitation is that the classification used preceded the development of ICD 11 and so can only be regarded as a very rough proxy of the new system. It is also fair to add that the interventions described in the study differ substantially and their impact was not planned with a common time scale. The populations treated were also very different and in future systematic reviews account will need to be made to the variation in personality domains at each severity level of personality disorder; it is not enough to assess severity alone. The procedure used in the analysis also did not follow the standard procedure for meta‐analytic reviews.

Nonetheless, there are several clear conclusions that can be drawn from the findings. First, it is clear from the data that the dimensional classification of personality is a reasonably robust and reliable indicator of severity of disorder. This represents a clear advance from previous classifications which have all relied on a dichotomous classification of disorder. Secondly, the presence of personality pathology is associated with predictably greater impairment of social functioning (Tyrer, Yang, et al. 2021) and the symptoms of anxiety and depression. This is clear evidence that personality pathology should not be ignored when a mental state examination is performed. The third finding is equivocal and is partly contradicted by the data. Previous work has suggested that the presence of significant personality pathology may have a braking effect on the outcome of those with anxiety and depressive disorders (Newton‐Howes et al. 2014; Skodol et al. 2014). Those with no personality pathology tend to respond much better to treatment and this improvement is maintained over the longer term. At the other end of the personality spectrum, those with moderate or severe personalities order are less likely to improve and maintain their progress over time.

The results of this study support the dimensional classification of personality pathology in ICD‐11 in some ways but not in others. Although consistent differences were found in anxiety pathology at different levels of ICD‐11 severity, this was not present for depressive symptoms and an expected difference of a worse outcome in those with personality disorder found in previous studies (Newton‐Howes et al. 2006, 2014) was not supported. An examination of the population differences offers a part explanation. The CHAMP study included a predominance of patients (medical patients) not seeking mental health treatment, with personality pathology in the domains of anankastia, dependence and negative affectivity with low levels of disinhibition. Those in the PEPS and POPMACT studies were treatment‐seeking and had problems of emotional instability, disinhibition and self‐harm, and those in the Nottingham study constituted predominantly mild but persistent mood disorders with all domains of personality represented more or less equally (Tyrer et al. 1990). Those with personality disorders in the CHAMP study had better outcomes in treatment than those with no personality disorder (Sanatinia et al. 2016; Tyrer, Wang, et al. 2021), probably related to better adherence to treatment, and so were very different to the other groups.

In future studies, the importance of the domain structure in ICD‐11 will need to be addressed. It will not be sufficient merely to classify by severity level only as populations at the same severity level can differ very greatly in their domain structure. A highly dissocial population is likely to be inconsistent in its adherence to treatment (Fonagy et al. 2025) compared with a treatment‐seeking medical one such as CHAMP (Tyrer, Wang, et al. 2021), and have very different outcomes in the longer term. The main advantage of the dimensional system is likely to be in the tailoring of interventions to the level of pathology, an exercise that has already been started but will need refinement (Grenyer et al. 2018; Lamph and Crawford 2024). There is also a need for the fine tuning of the pathological areas covered by each of the levels of severity, the most pressing of which is the need to set the boundaries of personality difficulty.

## Funding

Grants were received from the charity, NIDUS‐UK, and Imperial College for this study. We thank Mark Howells for assistance with data.

## Conflicts of Interest

The authors declare no conflicts of interest.

## Supporting information


**Data S1:** Follow‐up cases and missing data by outcomes.
**Data S2:** Comparison of baseline characteristics of individuals with and without missing data.
**Data S3:** Raw mean scores of outcomes by level of PD severity by treatment group.
**Data S4:** Raw mean scores of outcomes by follow‐up time in month.
**Data S5:** Raw Kendall Tau coefficients between ICD‐11 PD severity and outcomes by trial by treatment by follow‐up time and summary Kendall Tau coefficient by treatment by follow‐up time with 95% confidence intervals.
**Data S6:** Kendall Tau b coefficients by treatment by follow‐up time with 95% CI.
**Data S7:** Comparison of estimates of Model D between the model with MAR assumption and the model which removed cases with the most missing data during the follow‐ups in each trial.

## Data Availability

The data that support the findings of this study are available on request from the corresponding author. The data are not publicly available due to privacy or ethical restrictions.
