# Supplementary material for: An Individual Meta‐Analysis of Psychological Treatments for Mood and Personality Disorders Using the Severity Diagnostic System of ICD‐11
Source: Personal Ment Health. 2026 Oct 22;20(4):e70110. doi: 10.1002/pmh.70110 (PMC13691279; doi:10.1002/pmh.70110)
Supplement: Supplementary file 1 [file PMH-20-0-s001.docx]

**Supplementary Data**

**S1 Follow-up cases and missing data by outcomes**

| Trial | Follow-up period (month) | Depression  N: n (% missing) | Anxiety  N: n (% missing) | Social Function  N: n(%missing) |
| --- | --- | --- | --- | --- |
| NSND |  |  |  |  |
|  | Baseline | 157: 150 (4.5) | 157: 151 (3.8) | ---- |
|  | ≤ 4 | 157: 137 (12.7) | 157: 137 (12.7) | ---- |
|  | ≤ 7 | 157: 130 (17.2) | 157: 130 (17.2) | ---- |
|  | ≤ 12 | 157: 128 (18.5) | 157: 128 (18.5) | ---- |
|  | ≤25 | 157: 121 (22.9) | 157: 121 (22.9) | ---- |
| POPMACT |  |  |  |  |
|  | Baseline | 480: 426 (11.3) | 480: 425 (11.5) | 480: 426 (11.3) |
|  | ≤ 4 | ---- | ---- | ---- |
|  | ≤ 7 | 480: 427 (11.0) | 480: 427 (11.0) | 480: 427 (11.0) |
|  | ≤ 12 | 480: 415 (13.5) | 480: 415 (13.5) | 480: 428 (10.8) |
| CHAMP |  |  |  |  |
|  | Baseline | 444: 444 (0.0) | 444: 442 (0.45) | 444: 401 (9.7) |
|  | ≤ 4 | ---- | ---- | ---- |
|  | ≤ 7 | 444: 401(9.7) | 444: 401 (9.7) | 444: 380 (14.4) |
|  | ≤ 12 | 444: 386 (13.1) | 444: 384 (13.5) | 444: 372 (16.2) |
|  | ≤24 | 444: 370 (16.7) | 444: 370 (16.7) | 444: 365 (17.8) |
| PEPS |  |  |  |  |
|  | Baseline | 306: 306 (0.0) | 306: 304 (0.65) | 307: 305 (0.65) |
|  | ≤ 4 | 159: 157 (1.3) | 159: 155 (0.97) | 4: 4 (0.0) |
|  | ≤ 7 | 99: 99 (0.0) | 99: 98 (1.01) | 125: 123 (1.6) |
|  | ≤ 12 | 135: 135 (0.0) | 135: 135 (0.0) | 80: 77 (3.4) |
|  | ≤26 | 186: 185 (0.54) | 186: 186 (0.0) | 208: 203 (2.4) |
| All |  |  |  |  |
|  | Baseline | 1387: 1327 (4.1) | 1387: 1321 (4.6) | 1231: 1132 (8.0) |
|  | ≤ 4 | 313: 294 (6.1) | 313: 292 (6.7) | 4: 4 (0.0) |
|  | ≤ 7 | 1177: 1019 (13.4) | 1177: 1018 (13.5) | 1049: 930 (11.3) |
|  | ≤ 12 | 1213: 1078 (11.1) | 1213: 1076 (11.3) | 1004: 877 (12.6) |
|  | ≤26 | 784: 676 (13.8) | 784: 677 (13.6) | 652: 568 (12.9) |

Note：N = number of cases followed up; n = number of cases with outcome data. ---- No applicable

**S2** Comparison of baseline characteristics of individuals with and without missing data

|  | No missing | Miss one follow-up | Miss 2 follow-ups | Miss 3 follow-ups | Miss 4 follow-ups | Sig. P^a^ |
| --- | --- | --- | --- | --- | --- | --- |
| **NDSN** |  |  |  |  |  |  |
| N | 111-112 | 11 | 6 | 10 | 12 |  |
| Anxiety: Mean (SD) | 10.3 (5.4) | 11.1 (5.6) | 9.2 (2.9) | 10.8 (5.2) | 9.3 (4.6) | 0.847 |
| Depression: Mean (SD) | 14.0 (3.7) | 14.5 (4.5) | 13.8 (4.4) | 13.9 (4.4) | 11.5 (3.1) | 0.279 |
| Age: Mean (SD) | 38.1 (11.4) | 35.4 (11.9) | 26.2 (10.2) | 28.4 (8.4) | 33.8 (18.9) | 0.032 |
| Female: n (%) | 76 (67.9) | 10 (90.9) | 5 (83.3) | 6 (60.0) | 8 (66.7) | 0.370 |
| Active group: n (%) | 58 (51.8) | 8 (72.7) | 4 (66.7) | 7 (70.0) | 5 (41.7) | 0.561 |
| PD severity: n (%) |  |  |  |  |  | 0.389 |
| No PD | 50 (44.6) | 4 (36.4) | 3 (50.0) | 5 (50.0) | 2 (33.3) |  |
| PD difficulty | 23 (20.5) | 2 (18.2) | 2 (33.3) | 1 (10.0) | 0 (0) |  |
| Mild PD | 29 (25.9) | 2 (18.2) | 1 (16.7) | 2 (20.0) | 1 (16.7) |  |
| Moderate/severe PD | 10 (8.9) | 3 (27.3) | 0 (0) | 2 (20.0) | 3 (50.0) |  |
| **POPMACT** |  |  |  |  |  |  |
| N | 352-356 | 46-47 | 23-24 | ---- | ---- |  |
| Anxiety: Mean (SD) | 12.0 (4.9) | 10.6 (5.6) | 13.9 (3.4) | ----- | ---- | 0.000 |
| Depression: Mean (SD) | 9.0 (5.4) | 8.1 (5.3) | 11.2 (4.2) | **----** | **----** | 0.004 |
| Social function: Mean (SD) | 11.6 (4.9) | 10.5 (5.5) | 14.5 (3.6) | **----** | **----** | 0.006 |
| Age year: Mean (SD) | 32.4 (11.6) | 30.1 (10.4) | 26.3 (7.4) | **----** | **----** | 0.018 |
| Female: n (%) | 247 (70.2) | 36 (63.2) | 15 (62.5) | **----** | **----** | 0.561 |
| Active group: n (%) | 175 (49.7) | 29 (50.9) | 10 (41.7) | **----** | **----** | 0.684 |
| PD severity: n (%) |  |  |  |  |  | 0.573 |
| No PD | 34 (9.7) | 4 (7.0) | 2 (8.3) | **----** | **----** |  |
| PD difficulty | 169 (48.0) | 25 (43.9) | 13 (54.2) | **----** | **----** |  |
| Mild PD | 94 (26.7) | 21 (36.8) | 4 (16.7) | **-----** | **----** |  |
| Moderate/severe PD | 55 (15.6) | 7 (12.3) | 5 (20.8) | **----** | **----** |  |
| **PEPS** |  |  |  |  |  |  |
| N | 132, 160 | 24-25, 81 | 62-70 | 45-46 | 33 |  |
| Anxiety: Mean (SD) | 14.6 (3.9) | 14.3 (5.4) | 14.8 (3.5) | 15.2 (3.5) | 15.4 (3.5) | 0.681 |
| Depression: Mean (SD) | 12.9 (4.2) | 12.0 (5.7) | 12.6 (4.2) | 12.0 (4.7) | 13.2 (5.0) | 0.691 |
| Social function: Mean (SD) | 14.4 (4.2) | 14.7 (3.9) | 15.2 (4.9) | ---- | ---- | 0.447 |
| Age year: Mean (SD) | 39.9 (10.6) | 41.4 (11.3) | 36.2 (10.4) | 35.0 (10.8) | 39.1 (12.0) | 0.018 |
| Female: n (%) | 104 (78.8) | 18 (72.0) | 53 (75.7) | 37 (80.4) | 18 (54.5) | 0.051 |
| Active group: n (%) | 74 (56.1) | 15 (60.0) | 39 (55.7) | 20 (43.5) | 6 (18.2) | 0.002 |
| PD severity: n (%) |  |  |  |  |  | 0.518 |
| No PD | ---- | ---- | ---- | ---- | ---- |  |
| PD difficulty | 21 (15.9) | 3 (12.0) | 11 (15.7) | 6 (13.0) | 7 (21.2) |  |
| Mild PD | 13 (39.4) | 8 (32.0) | 33 (47.1) | 15 (32.6) | 13 (39.4) |  |
| Moderate/severe PD | 13 (39.4) | 14 (56.0) | 26 (37.1) | 25 (54.3) | 13 (39.4) |  |
| **CHAMP** |  |  |  |  |  |  |
| N | 276, 340 | 52, 70 | 23-24 | 29 | ---- |  |
| Anxiety: Mean (SD) | 12.3 (3.7) | 13.4 (3.8) | 12.6 (4.1) | 11.4 (4.3) | ---- | 0.115 |
| Depression: Mean (SD) | 9.3 (4.1) | 9.3 (4.1) | 9.7 (5.3) | 6.8 (3.3) | ---- | 0.041 |
| Social function: Mean (SD) | 9.6 (4.4) | 9.3 (4.3) | 10.8 (4.8) | 7.7 (4.9) | ---- | 0.083 |
| Age year: Mean (SD) | 47.4 (13.0) | 47.4 (13.0) | 45.1 (14.9) | 50.1 (13.4) | ---- | 0.479 |
| Female: n (%) | 183 (53.8) | 27 (51.9) | 13 (56.5) | 13 (44.8) | ---- | 0.804 |
| Active group: n (%) | 172 (50.6) | 23 (44.2) | 10 (43.5) | 14 (48.3) | ---- | 0.792 |
| PD severity: n (%) |  |  |  |  | ---- | 0.807 |
| No PD | 49 (14.4) | 6 (11.5) | 2 (8.7) | 6 (20.7) | ---- |  |
| PD difficulty | 154 (45.3) | 20 (38.5) | 9 (39.1) | 14 (48.3) | ---- |  |
| Mild PD | 105 (30.9) | 20 (38.5) | 9 (39.1) | 8 (27.6) | ---- |  |
| Moderate/severe PD | 32 (9.4) | 6 (11.5) | 3 (13.0) | 1 (3.4) | ---- |  |

a One-way ANOVA test for anxiety, depression and age, Fisher’s Exact test for other variables.

---- Not applicable

**S3** Raw mean scores of outcomes by level of PD severity by treatment group

| Trial  PD level | Depression  Mean (SD) | |  | Anxiety  Mean (SD) | |  | Social function  Mean (SD) | |
| --- | --- | --- | --- | --- | --- | --- | --- | --- |
|  | Active | Control |  | Active | Control |  | Active | Control |
| NSND |  |  |  |  |  |  |  |  |
| Lev. 1 | 7.1 (5.1) | 5.3 (4.7) |  | 9.5 (5.4) | 9.5 (5.7) |  | ----- | ----- |
| Lev. 2 | 6.8 (5.1) | 7.3 (4.6) |  | 9.6 (5.6) | 11.1 (3.9) |  | ----- | ----- |
| Lev. 3 | 8.7 (5.5) | 8.6 (4.7) |  | 12.4 (5.1) | 11.0 (4.2) |  | ----- | ----- |
| Lev. 4 | 10.3 (5.6) | 8.3 (5.2) |  | 11.3 (5.8) | 9.4 (5.6) |  | ----- | ----- |
| POPMACT |  |  |  |  |  |  |  |  |
| Lev. 1 | 7.5 (5.3) | 7.4 (5.1) |  | 10.6 (4.4) | 10.9 (4.5) |  | 9.7 (4.7) | 10.0 (4.0) |
| Lev. 2 | 8.2 (5.3) | 8.3 (5.2) |  | 11.2 (5.0) | 11.1 (5.1) |  | 10.5 (5.0) | 10.7 (5.0) |
| Lev. 3 | 9.4 (5.5) | 9.0 (4.0) |  | 12.6 (5.0) | 12.5 (4.5) |  | 11.9 (5.1) | 12.2 (4.7) |
| Lev. 4 | 11.1 (5.4) | 10.7 (5.3) |  | 14.2 (5.7) | 14.3 (3.8) |  | 14.1 (5.1) | 13.9 (4.2) |
| CHAMP |  |  |  |  |  |  |  |  |
| Lev. 1 | 7.3 (4.5) | 6.0 (4.3) |  | 9.7 (3.9) | 9.2 (4.3) |  | 7.2 (4.3) | 6.1 (4.3) |
| Lev. 2 | 7.2 (4.4) | 7.8 (4.2) |  | 10.0 (4.5) | 10.6 (4.1) |  | 7.7 (4.7) | 8.4 (4.4) |
| Lev. 3 | 9.1 (4.7) | 9.2 (4.4) |  | 11.1 (4.6) | 12.0 (4.2) |  | 10.7 (5.4) | 10.6 (4.6) |
| Lev. 4 | 9.5 (4.6) | 11.4 (4.9) |  | 11.3 (4.7) | 12.6 (4.5) |  | 11.4 (4.5) | 12.7 (5.0) |
| PEPS |  |  |  |  |  |  |  |  |
| Lev. 1 | ----- | ----- |  | ----- | ----- |  | ----- | ----- |
| Lev. 2 | 10.9 (4.4) | 9.9 (5.3) |  | 12.9 (4.3) | 12.2 (4.5) |  | 11.6 (5.1) | 12.3 (4.8) |
| Lev. 3 | 11.1 (4.9) | 11.6 (4.8) |  | 13.9 (3.8) | 14.3 (4.1) |  | 14.2 (4.4) | 14.0 (4.3) |
| Lev. 4 | 12.5 (4.9) | 13.3 (4.9) |  | 14.2 (4.1) | 15.2 (4.1) |  | 15.0 (4.3) | 15.0 (4.2) |

Note: Lev. 1 for No PD, Lev. 2 for PD Difficult, Lev. 3 for Mild PD, Lev. 4 for Moderate and Severe PD. ----- Not applicable. **S4** Raw mean scores of outcomes by follow-up time in month

| Trial | Depression | Anxiety | Social function |
| --- | --- | --- | --- |
|  | Mean (SD) | Mean (SD) | Mean (SD) |
| NSND |  |  |  |
| Baseline | 10.3 (4.5) | 13.8 (3.8) | ---- |
| 1-4 | 7.1 (4.5) | 10.2 (5.2) | ---- |
| 5-7 | 6.6 (5.2) | 9.4 (5.4) | ---- |
| 8-12 | 6.6 (5.4) | 9.3 (5.1) | ---- |
| 13-26 | 5.7 (5.3) | 8.4 (5.0) | ---- |
| POPMACT |  |  |  |
| Baseline | 9.0 (5.4) | 11.9 (5.0) | 11.6 (5.0) |
| 6 | 9.3 (5.4) | 12.4 (5.0) | 11.8 (5.0) |
| 12 | 8.2 (5.1) | 11.5 (4.8) | 10.9 (5.0) |
| CHAMP |  |  |  |
| Baseline | 8.8 (4.2) | 12.4 (3.8) | 9.4 (4.5) |
| 6 | 8.0 (4.5) | 10.4 (4.5) | 9.2 (5.2) |
| 12 | 8.0 (4.8) | 10.2 (4.4) | 8.9 (5.1) |
| 24 | 7.9 (5.0) | 9.7 (4.6) | 8.5 (5.1) |
| PEPS |  |  |  |
| Baseline | 12.4 (4.5) | 14.7 (3.7) | 14.7 (4.3) |
| 1-4 | 11.9 (5.3) | 14.0 (4.3) | 11.0 (7.1) |
| 5-7 | 11.3 (4.6) | 13.1 (4.2) | 13.5 (4.3) |
| 8-12 | 11.3 (4.9) | 13.9 (4.1) | 13.7 (4.5) |
| 13-26 | 10.9 (5.3) | 13.3 (4.6) | 13.8 (4.8) |

Note: ---- Not applicable

**S5 Raw Kendall Tau coefficients between ICD-11 PD severity and outcomes by trial by treatment by follow-up time and summary Kendall Tau coefficient by treatment by follow-up time with 95% confidence intervals**

|  | Depression |  | Anxiety |  | Social function |
| --- | --- | --- | --- | --- | --- |
| Subgroups | n: Tau (95%CI) |  | n: Tau (95%CI) |  | n: Tau (95%CI) |
| Baseline NSND Control | 68: 0.128 (-0.067, 0.322) |  | 69: -0.209 (-.395, -.024) |  | N/A |
| Baseline NSND Active | 82: 0.177 (0.005, 0.349) |  | 82: 0.092 (-0.074, 0.258) |  | N/A |
| Baseline POPMACT Control | 216: 0.083 (-0.02,0.187) |  | 217: 0.188 (0.088, 0.288) |  | 217: 0.192 (0.103, 0.282) |
| Baseline POPMACT Active | 209: 0.157 (0.055, 0.26) |  | 209: 0.161 (0.058, 0.265) |  | 209: 0.165 (0.063, 0.267) |
| Baseline CHAMP Control | 224: 0.255 (0.155, 0.355) |  | 225: 0.239 (0.15, 0.328) |  | 210: 0.341 (0.244, 0.439) |
| Baseline CHAMP Active | 218: 0.294 (0.197, 0.391) |  | 219: 0.224 (0.123, 0.324) |  | 191: 0.431 (0.353, 0.51) |
| Baseline PEPS Control | 153: 0.157 (0.037,0.277) |  | 152: 0.167 (0.044, 0.29) |  | 154: 0.145 (0.020, 0.265) |
| Baseline PEPS Active | 155: 0.140 (0.023, 0.257) |  | 152: 0.167 (0.044, 0.29) |  | 151: 0.181 (0.058, 0.303) |
| 0.6-3 months PEPS Control | 91: 0.164 (0.025, 0.302) |  | 90: 0.209 (0.047, 0.371) |  | N/A |
| 0.6-3 months PEPS Active | 112: 0.222 (0.087, 0.357) |  | 111: 0.008 (-0.147, 0.164) |  | N/A |
| 3-4 months NSND Control | 63: 0.20 (0.011, 0.389) |  | 63: 0.071 (-0.151, 0.292) |  | N/A |
| 3-4 months NSND Active | 74: 0.046 (-0.133, 0.225) |  | 74: 0.053 (-0.112, 0.218) |  | N/A |
| 6 months POPMACT Control | 213: 0.148 (0.044, 0.252) |  | 213: 0.135 (0.036, 0.233) |  | 212: 0.159 (0.059, 0.26) |
| 6 months POPMACT Active | 214: 0.146 (0.046, 0.246) |  | 214: 0.184 (0.084, 0.284) |  | 212: 0.259 (0.164, 0.353) |
| 4-6 months PEPS Control | 44: 0.25 (0.014, 0.485) |  | 44: 0.252 (0.047, 0.456) |  | 42: -0.09 (-0.320, 0.140) |
| 4-6 months PEPS Active | 58: 0.002 (-0.227, 0.231) |  | 57: -0.011 (-0.222, 0.199) |  | 28: 0.347 (0.087, 0.608) |
| 6 months CHAMP Control | 204: 0.26 (0.157, 0.364) |  | 204: 0.199 (0.09, 0.307) |  | 197: 0.292 (0.186, 0.399) |
| 6 months CHAMP Active | 197: 0.139 (0.032, 0.246) |  | 197: 0.05 (-0.056, 0.156) |  | 183: 0.185 (0.077, 0.293) |
| 7 months NSND Control | 60: 0.21 (0.003, 0.416) |  | 60: 0.06 (-0.153, 0.274) |  | N/A |
| 7 months NSND Active | 70: 0.17 (-0.0002, 0.341) |  | 70: 0.137 (-0.028, 0.301) |  | N/A |
| 12 months POPMACT Control | 207: 0.156 (0.053, 0.26) |  | 207: 0.219 (0.122, 0.315) |  | 207: 0.206 (0.113, 0.30) |
| 12 months POPMACT Active | 208: 0.161 (0.053, 0.27) |  | 208: 0.219 (0.118, 0.321) |  | 208: 0.157 (0.053, 0.261) |
| 7-12 months PEPS Control | 64: 0.152 (-0.035, 0.34) |  | 64: 0.174 (-0.024, 0.371) |  | 70: 0.191 (0.011, 0.371) |
| 7-12 months PEPS Active | 72: 0.116 (-0.057, 0.291) |  | 72: 0.127 (-0.063, 0.316) |  | 64: 0.112 (-0.087, 0.31) |
| 12 months CHAMP Control | 192: 0.221 (0.109, 0.333) |  | 192: 0.202 (0.089, 0.315) |  | 185: 0.287 (0.175, 0.399) |
| 12 months CHAMP Active | 192: 0.131 (0.018, 0.244) |  | 194: 0.078 (-0.034, 0.19) |  | 187: 0.208 (0.101, 0.315) |
| 12 months NSND Control | 58: 0.303 (0.112, 0.494) |  | 58: 0.275 (0.078, 0.472) |  | N/A |
| 12 months NSND Active | 70: 0.137 (-0.04, 0.313) |  | 70: 0.215 (0.055, 0.374) |  | N/A |
| 13-26 months PEPS Control | 83: 0.302 (0.147, 0.458) |  | 84: 0.245 (0.074, 0.416) |  | 110: 0.185 (0.040, 0.331) |
| 13-26 months PEPS Active | 102: 0.167 (0.036, 0.298) |  | 102: 0.13 (-0.027, 0.289) |  | 93: 0.224 (0.074, 0.374) |
| 24 months CHAMP Control | 181: 0.227 (0.108, 0.345) |  | 181: 0.13 (0.014, 0.246) |  | 180: 0.304 (0.196, 0.411) |
| 24 months CHAMP Active | 189: 0.04 (-0.07, 0.152) |  | 189: 0.105 (-.001, 0.211) |  | 185: 0.156 (0.055, 0.257) |
| 24 months NSND Control | 56: 0.422 (0.279, 0.565) |  | 56: 0.241 (0.06, 0.423) |  | N/A |
| 24 months NSND Active | 65: 0.249 (0.071, 0.426) |  | 65: 0.261 (0.091, 0.432) |  | N/A |
| **Overall Control** | 671: 0.194 (0.149, 0.239) |  | 673: 0.173 (0.128, 0.218) |  | 581: 0.220 (0.145, 0.294) |
| **Overall Active** | 700: 0.161 (0.108, 0.214) |  | 700: 0.138 (0.086, 0.190) |  | 551: 0.208 (0.137, 0.279) |
| **Baseline overall** | 1371: 0.182 (0.128, 0.236) |  | 1373: 0.159 (0.106, 0.212) |  | 1132: 0.244 (0.154, 0.334) |
| **1- 4 months overall** | 315: 0.164 (0.072, 0.256) |  | 313: 0.083 (-.009, 0.175) |  | N/A |
| **5- 7 months overall** | 1158: 0.169 (0.10, 0.238) |  | 1157: 0.135 (0.066, 0.204) |  | 874: 0.20 (0.104, 0.296) |
| **8-12 months overall** | 1102: 0.168 (0.099, 0.237) |  | 1105: 0.184 (0.115, 0.253) |  | 921: 0.196 (0.10, 0.292) |
| **13-26 months overall** | 518: 0.171 (0.095, 0.247) |  | 518: 0.160 (0.085, 0.234) |  | 563: 0.20 (0.096, 0.304) |

**S6 Kendall Tau b coefficients by treatment by follow-up time with 95% CI**

**S7 Comparison of estimates of Model D between the model with MAR assumption and the model which removed cases with the most missing data during the follow-ups in each trial**

|  | Depression | |  | Anxiety | |
| --- | --- | --- | --- | --- | --- |
| Variable | Main analysis under MAR | Sensitive cases removed |  | Main analysis under MAR | Sensitive cases removed |
|  | Mean (SE)  N=1374 | Mean (SE)  N=1288 |  | Mean (SE)  N=1375 | Mean (SE)  N=1288 |
| Active (vs Control) | -0.155 (0.539) | -0.199 (0.560) |  | -0.391 (0.493) | -0.504 (0.513) |
| Follow-up month | -0.058 (0.026) | -0.058 (0.026) |  | -0.070 (0.026) | -0.068 (0.026) |
| Active * month | -0.042 (0.036) | -0.041 (0.036) |  | -0.025 (0.035) | -0.021 (0.035) |
| PD (vs Moderate/severe) |  |  |  |  |  |
| No PD | -3.916 (0.634) | -3.991 (0.660) |  | -2.296 (0.577) | -2.152 (0.602) |
| PD difficulty | -3.036 (0.488) | -3.155 (0.508) |  | -2.438 (0.446) | -2.426 (0.466) |
| Mild PD | -1.814 (0.496) | -1.869 (0.519) |  | -0.902 (0.453) | -0.969 (0.476) |
| Active * PD |  |  |  |  |  |
| No PD | 0.695 (0.857) | 0.827 (0.882) |  | 0.033 (0.781) | 0.083 (0.807) |
| PD difficulty | -0.054 (0.670) | -0.042 (0.69)3 |  | 0.799 (0.613) | 0.857 (0.636) |
| Mild PD | 0.466 (0.683) | 0.541 (0.706) |  | 0.321 (0.625) | 0.597 (0.648) |
| Month* PD |  |  |  |  |  |
| No PD | -0.076 (0.036) | -0.075 (0.036) |  | -0.118 (0.035) | -0.122 (0.035) |
| PD difficulty | 0.015 (0.031) | 0.017 (0.031) |  | 0.008 (0.031) | 0.007 (0.031) |
| Mild PD | 0.023 (0.032) | 0.023 (0.032) |  | 0.009 (0.031) | 0.010 (0.032) |
| Active*month*PD |  |  |  |  |  |
| No PD | 0.096 (0.049) | 0.093 (0.050) |  | 0.085 (0.049) | 0.083 (0.049) |
| PD difficulty | 0.021 (0.043) | 0.021 (0.043) |  | -0.049 (0.042) | -0.051 (0.042) |
| Mild PD | -0.018 (0.043) | -0.020 (0.044) |  | -0.020 (0.043) | -0.028 (0.043) |
